# Class-Room Talks No. 7

**Published:** 1888-09

**Authors:** J. T. Kent


### CLASS-ROOM TALKS. No. 7.

(From the Lectures of Professor J. T. Kent, M. D.)

Upon the subject of dietetics—the assimilation of the necessary elements of the great macrocosm that go to form the microcosm of physical man; the food from which these elements are gathered by the animal economy; the difference in amount of such elements required in individual cases, and how regulated; the inanition for those elements; the cause; their treatment, and cure—I would like to have a word with you.

There are, at the present time, so many papers coming out on this grave subject, covering it only from an external point of view, that you have need that somebody show you wherein that which sounds so plausible is *untrue*, and wherein it is both true and useful.

When you find, in child or adult, a manifest inanition for some element—be it bone-salt, Sulphur, Nat-mur., or other of the necessary substances—recognize this possibility. There may be at least three different sources from which to look for the cause which has exhibited this condition; that looking at *one*, and that the most *infrequent*, will not give you the success, which, as homœopaths, you have been led to expect.

1st. As an external cause, you *may* find that the proper element does *not* enter—as a constituent or in sufficient quantities—into the ordinary food the patient takes. A condition comparatively *sure* and certainly seen only in those whom poverty has deprived of the commonest necessities of life.

2d. You will find those in whom there is an *inability* to assimilate or appropriate those elements, no matter in what abundance they may have been supplied. See cases of inanition for Natr-mur. in which any amount of table salt has been craved and eaten for years, perhaps with tenacious eating and constant emaciation, or an inanition for Calcaria or bone-salts, which, if fed to them by the pint, would only aggravate the condition, torment the patient, and not hasten the growth of a single tooth, or add strength to one of the softening bones.

3d. We may find a combination of the two causes, insufficient food and inefficient assimilation, *both* of which you must take the proper means to correct.

The true reason for this want of assimilation lies in the disturbance of the vital force by some miasm. If you know the parents of a child to have been deeply psoric, and if it has not been wise to disturb that miasm in the mother during the period of gestation, with the properly selected remedy, give to the child, at birth, the dose, and before another pregnancy place the parents in better condition. You will be astonished to find how satisfactorily the process of bone formation and dentition will go on, and if you compare the result with such as you have had before, wherein you have made the selection of food of the finest quality and exact quantity, you will find that in the majority of cases the latter has only acted as what our neighbors of the "regular" school would call an excellent adjuvant. How much, think you, would the best dietetic regimen do to prevent the blackening, crumbling, and decaying of teeth, the gradual destruction of bone tissue—as in hip-joint disease—if a syphilitic miasm were inherited?

I suspect all measures of that kind would be positively evil without the properly selected remedy.

In the matter of assimilation of the natural elements physiology teaches that the human economy in its several divisions *takes* that which it needs from the blood, and we only supply nutrition, then placing it in the proper receptacle, from which it is extracted in quantities and qualities to suit the individual needs of the different parts of the system.

*We* do not send certain per cent. of Calc., Phos., or Sil. to the bones per day, or of other constituents of the body to the muscles, nerves, and cartilege. We simply send a supply, greater or less, *en masse,* which nature, if normal, regulates by the appetite, and which the tissues *themselves* again regulate according to their need. Greater waste or use having been made at one time than another, therefore greater demand. So, then, in pathological conditions, of what use a choice of food containing 0.645 of Lime and 0.874 of Phos.-ac. if the system, because of the miasmatic disturbances can only absorb and assimilate 0.001 of it.

You may think it well to place it there in case of emergency

—it might be used. Doubted. There is such a thing as creating a poisoning by forced use of a substance that causes even greater depression of the vital force than would have been done by nature herself. A careful study of the miasmatic aspect and a careful application of the indicated remedy will give nature the chance to regulate her own supplies better than you can possibly regulate them for her.

The substances of the inorganic world can only be assimilated and made of use to the animal economy through their rehabilitation in the vegetable kingdom. When entered in the crude they simply form obstruction to the normal functions of the vital force, throwing out their own signs and symptoms, acting as an irritant or foreign body, and if left unmolested causing death with greater or less rapidity, according to the vitality of the organ or organs which they obstruct or to the completeness of the obstruction.

The differences of requirement perfectly normal of the different tissue builders in individual cases is greatly varied, how then must it be in pathological conditions? How else can we determine except by signs and symptoms? There is one very good way to tell if it is the food that disagrees with your patient, and that is just this, if the patient *ever has thriven* upon the diet which now disagrees you may be sure that it is a remedy that is needed and *not* a change of food.

In one of our late papers upon dietetics the author remarks that the "teeth once built up are built up forever." One of the finest looking, and the dentists said, the soundest set of teeth I ever saw belonged to a young woman of twenty-four. Before she was thirty-five years of age she had lost them, and had a complete artificial set, they had quite crumbled away, and had had good care. Query, is good care necessary to the preservation of teeth in a good, healthy organism? A son of the above-mentioned subject was fed upon cereals during his baby life, in fact, would eat nothing else. He lost his teeth by decay long before it was time for the second set. Another son had a great craving for milk, would hardly be persuaded to eat other things when seven or eight years old. He had good teeth so long as I knew of him. What was the matter there?

I wish to show you this, that you may look for the *deeper cause* in your efforts to bring into this world of ours a more healthy and righteous condition of things. Do not stop to weigh and measure the food put into the body until you find whether the force occupying that body is capable of assimilating those elements to its own best good. Search first for the remedy and then for proper food, remembering that it matters not the quantity or quality *if* the economy absolutely refuses to make use of it. S. L. G. L.

