# Errata

**Published:** 1888-10

**Authors:** 


ERRATA.—In September issue, page 476, sixth line from bottom, for *sure* read *rare;* page 475, for *tenacious* read *voracious;* line fourteenth from bottom, for *then* read *by*.

